# Isolation and Identification of Non- Commensal Pathogenic Bacteria in the Saliva of Patients Candidate for Liver Transplant: A Cross Sectional Study in Shiraz, South of Iran

**DOI:** 10.30476/DENTJODS.2019.77854.

**Published:** 2020-06

**Authors:** Jannan Ghapanchi, Abdollah Bazargani, Hooman Khorshidi, Maryam Erfani, Fahimeh Rezazadeh, Azita Azad, Reza Derafshi, Ahmad Hassan Kalantari

**Affiliations:** 1 Dept. of Oral and Maxillofacial Medicine, School of Dentistry, Shiraz University of Medical Sciences, Shiraz, Iran; 2 Dept. of Bacteriology and Virology, School of Medicine, Shiraz University of Medical Sciences, Shiraz, Iran; 3 Dept. of Periodontics, School of Dentistry, Shiraz University of Medical Sciences, Shiraz, Iran; 4 Student, School of Dentistry, Shiraz University of Medical Sciences, Shiraz, Iran; 5 Oral & Dental Disease Research Center, Dept. of Oral and Maxillofacial Medicine, School of Dentistry, Shiraz University of Medical Sciences, Shiraz, Iran; 6 Dept. of Prosthetics, Biomaterials Research Center, School of Dentistry, Shiraz University of Medical Sciences, Shiraz, Iran

**Keywords:** Gram negative bacteria, Saliva, Hepatic disorder

## Abstract

**Statement of the Problem:**

Liver cirrhosis is the end stage of liver failure. It is mentioned as one of the main etiologies of morbidity and mortality in the world. The human salivary bacteria may induce oral disorders and interact with other body microbiota.

**Purpose:**

The aim of the present study is to identify the pathogenic bacteria of non-oral origin from the saliva samples of patients with end stage liver failure.

**Materials and Method:**

In this cross-sectional study, the saliva samples of 88 end stage liver disease cases and 84 age- and sex-matched healthy subjects were collected. The samples were cultured using gram staining and API20E Kit.

**Results:**

According to the statistical analysis, the total amount of the non-commensal bacteria was significantly higher in chronic liver failure (CLF) group than controls (*p*= 0.001). There was no significant difference between both groups for the presence of other bacteria (*p*= 0.001) except for *Escherichia coli* (*E. coli*). *E. coli* was isolated from the saliva of 15 cases and only 2 controls.

**Conclusion:**

Oral cavity may act as a reservoir for enteric bacteria such as *E. coli* in liver failure patients. Adequate oral and general hygiene might reduce the risk of systemic infection especially in immunocompromised cases.

## Introduction

Liver cirrhosis is the end stage of liver failure and mentioned as one of the main etiologies of morbidity and mortality in the world [ 1 ]. Many diseases especially viral hepatitis, alcoholic liver disease and nonalcoholic fatty liver disease/nonalcoholic steatohepatitis, may lead to cirrhosis. Currently, viral hepatitis (hepatitis C and B) is the main etiology of liver failure; however, nonalcoholic fatty liver disease/ nonalcoholic steatohepatitis is estimated to become another important cause of chronic liver disease, particularly in diabetic subjects [ 2 ]. Cirrhosis may cause a pro-inflammatory situation that can enhanced disease development and complications like hepatic encephalopathy and infections [ 3 ]. Infectious diseases are main etiologies for morbidity and mortality in end stage liver failure and transplant recipient. Oral cavity infection is a source of general infection for large numbers of liver transplant candidates and recipients. Liver transplant cases are at higher risk of oral infection and protective methods are essential [ 4 ]. There is a sturdy relation between the gut microbiota and liver cirrhosis consequences, a relationship that requires more examinations [ 5 ]. It is still unclear, if this dysbiosis-inflammatory status occurs only in the gut or is a widespread phenomenon in cirrhosis. Comparable to the effect of gut bacteria on cirrhosis, new evidence also proposes that there is a conceivable link between a dysbiotic oral environment and liver failure [ 6 ]. Qin *et al*. [ 7 ] reported that oral bacteria could exist in the stool but the direct assessment of the oral microbiota has not been examined in liver cirrhosis. The study of salivary protection is imperative in a microbiota-immune alteration as salivary bacteria might affect the distal gut microbiota [ 8 ]. The human salivary microbiota can interact with other parts of the body microflora especially the intestinal tract, however little is identified about normal dissimilarity in the salivary bacteria [ 8 - 9 ]. Relatively, little consideration has been paid to the human salivary bacteria, as most researches have focused on finding microbiota that might be related to oral diseases [ 10 - 11 ].

The aim of the present study is to isolate the pathogenic bacteria of non-oral origin from the saliva samples of patients with end stage liver failure.

## Materials and Method

### Ethical Statement

This study was carried out in accordance with the guidelines of the Declaration of Helsinki as revised in Edinburgh (1975). The study protocol was approved by the Ethics Committee of Shiraz University of Medical Sciences, Shiraz, Iran. The written informed consents were obtained from all participants for sample collection and in disabled cases verbal consent was obtained. All patients were informed about the nature of the study and subjects with no desire to participate in the research were excluded.

### Reagents

Eosin methylene blue, thioglycollate broth, blood agar, barium chloride, sulfuric acid, crystal violate, Safranin, lugol solution, acetone, ethanol, oxidase and catalase reagents were purchased from Merck (Germany). API20E kit was obtained from Biomerieux (France). All other chemicals were commercially available.

### Participants

In a cross sectional study (June to December 2017), salivary samples of 88 end stage liver disease cases who attended Imam Reza subspecialized clinic (Shiraz, South west Iran), were collected. All samples were collected between 10-12 am. The study group comprised of 60 males and 28 females who were candidate for liver transplant (known cases of liver cirrhosis based on Child Pugh and MALT criteria). A dentist should visit all chronic liver failure (CLF) patients prior to transplant in order to eliminate oral infective sources. The control group included 84 age- and sex-matched healthy participants referred to various departments of Shiraz dental school for routine dental care. The cases and control group also matched according to tooth brushing frequency. The subjects were selected according to age, gender and absence of any systemic disease. Patients with clinical evidence of oral mucosal lesions, history of systemic disease such as diabetes mellitus, smoking, pregnancy, use of antimicrobial mouthwash, or treatment with antibiotics in the past two months were excluded from the study. Demographic data and the oral health status of the participants were recorded.

### Saliva sampling and microbial culture

The unstimulated whole saliva (UWS) [ 12 ] was collected at least 60 minutes after the last intake of drink or food. The subjects were instructed to spit 3 ml UWS into sterile Falcon tubes containing 1 ml normal saline. Every contributor was asked to refrain from eating and drinking one hour before sampling. Afterward their lips were cleaned and each one rinsed his/her mouth with water, collection was done by a general dentist. The collected samples were sent to the microbiology department of medical school affiliated with Shiraz University of Medical Sciences.

Each sample was centrifuged at 12,500 rpm for 10 min and the supernatant was discarded. The precipitate was suspended in 1ml of phosphate-buffered saline to obtain a concentrated sample suspension. One loop full of concentrated suspension was inoculated onto eosin methylene blue and MacConkey agar culture media utilizing a standard streak plate method. All culture plates were incubated at 37°C for 24 h, and the growth of bacteria was perceived as pink- and white-colored colonies. The suspected colonies were exposed to Gram stain to detect gram-negative rod bacteria. Once detected, the colonies were further subjected to biochemical reactions by API20E Kit (Biomerieux, France) [ 13 ].

### Statistical analysis

All data were analyzed using SPSS software version 23. The Fisher exact test with odds ratio (95% confidence interval) was used to correlate the positive and negative cases with the disease. Student t test was used to compare the groups regarding age. Statistically significant difference was considered when *p*< 0.05.

## Results

The age in case group ranged from 18 to 66 years (mean age: 40.99±15.55 years), and in the control from 16 to 68 years (mean age: 36.9 ±10.6 years). 

According to statistical analysis, the total amount of the non-commensal bacteria was significantly higher in CLF cases (*p*= 0.001). Forty seven (53.4%) of the cases were negative for contamination with non-oral pathogenic bacteria and 41(46.6%) were positive. In contrast in the control group, 71(84.5%) were negative and 13 (15.5%) were positive (*p*= 0.0001). The saliva samples of case group exhibited more than one type of non-commensal bacteria including, *Klebsiella pneumoniae, Enterobacter cloacae, Acinetobacter sp, Raoultella sp, Pseudomonas aeruginosa, Providencia sp, Serratia sp*.

 Individually except for *Escherichia coli* (*E. coli*) isolate (*p*= 0.001), there was no significant difference between both groups for the presence of other bacteria. *E. coli* isolated from the saliva of 15 cases and only 2 controls. There was no significant correlation between age and presence of bacteria in the oral cavity (*p*= 0.516). There was no significant correlation between gender and presence of the non-oral pathogens (*p*= 0.70) (Table 1). There was no significant correlation between tooth brushing, frequency and presence of the bacteria in both case and controls (*p*= 0.253) (Table 2).

**Table 1:** Salivary detection of non-oral bacteria from healthy and CLF patients

Parameters		CLF (%)	Control (%)	Total	*p* Value
**Age**		40.24	41.79		0.516
Sex	Female	28	38		0.7
	Male	60	46		
Non commensal Bacteria	+	41(46.6%)	13(15.5%)	54	0.000
	-	47(53.4%)	71(84.5%)	118	
	Total	88(100%)	84(100%)	172	
E. coli	+	15(17.0%)	2(2.4%)	17	0.001
	-	73(83.0%)	82(97.6%)	155	
	Total	88(100%)	84(100%)	172	

p= 0.001- Chronic liver Failure cases (CLF)

**Table 2:** Relation between non commensal bacteria and tooth brushing frequency

			Non commensal Bacteria		Total
			Negative	Positive	
Brushing	**Once**	**Count**	46	14	60
		% within Brushing	76.7%	23.3%	100.0%
	Twice	Count	19	12	31
		% within Brushing	61.3%	38.7%	100.0%
	Three	Count	8	2	10
		% within Brushing	80.0%	20.0%	100.0%
	None	Count	45	26	71
		% within Brushing	63.4%	36.6%	100.0%
Total		Count	118	54	172
		% within Brushing	68.6%	31.4%	100.0%

## Discussion

In the past few years, the progression of medical technologies directed to amazing findings about the human microbial strains. The human intestine inhabits trillions of bacteria; several of them are metabolically active. Both host and environmental factors influence microbiome virulence. A study by Almerich *et al*. [ 14 ] showed that oral anatomical and physiological properties make it a favorite location for bacterial growth. The saliva or oropharyngeal secretions have a significant role in bacterial spreading through sneezing, coughing, speaking, or breathing.

In the present study, the difference for microbial population in saliva samples between participants with end stage liver disease and the healthy group was compared showing a variety of pathogens in these cases including, *Klebsiella pneumonia, Enterobacter cloacae, Acinetobacter sp, Raoultella sp, Pseudomonas aeruginosa, Providencia sp, Serratia sp*.

It is accepted that systemic or oral condition alterations can affect the mouth microflora [ 9 ]. On the other hand, pathologic oral microbiota can influence many important systemic diseases.

Numerous factors can affect the oral microbiota colonization such as hospitalization, immune status alteration, inadequate oral hygiene, xerostomia and jaw movement that can improve Enterobacteriaceae colonization [ 15 ]. Recently, numerous studies have concentrated on the correlation between periodontal diseases, oral microflora and systemic diseases [ 16 ].

Animal-based trials showed that periodontitis might contribute in the development of hepatic diseases, such as non-alcoholic fatty liver disease, cirrhosis and hepatocellular carcinoma; in addition, it may also influence liver transplantation [ 17 ]. 

Since gingivitis or periodontitis are usual oral disorders experienced by liver transplant cases, it is essential to differentiate whether an identified bacterial alteration is derived from the general immune status or by current periodontal disease [ 18 ].

Bajaj *et al*. [ 19 ] reported sign of prominent immune-microbiota communication alteration in the saliva and stool of cirrhotic patients. This phenomenon is related to inflammation, variations in bacterial defenses and consequent liver-related hospitalizations.

There was extensive inflammation associated with Th1 and Th17 system stimulation in the blood circulation of cirrhotic cases, particularly those with prior hepatic encephalopathy [ 20 ].

The cirrhotic group showed a pro-inflammatory state in the saliva with increased level of IL-1β and IL-6 concentration and a subsequent rise in secretory IgA. This co-existed with reduced innate local defenses and decreased histatins 1 and 5 and lysozyme. In CLF cases, higher fecal secretory IgA secreted into saliva and initiate systemic inflammation, possibly through contributors in the intestine and the oral fossa [ 21 ].

The bacterial flora of the tongue surface was also evaluated. It is estimated that 43% of the population had *Enterobacter* and *pseudomonas* on the tongue dorsum, which was more common in the age range of 40–50 years and nonsmokers. This result represents that tongue surface might a first pool of the microbiota [ 22 ].

It seems that non-commensal bacteria in immune competent subjects are not a main concern, although in immunosuppressed cases is a hazardous pathogen.

Based on our results, *E. coli* in cirrhotic patients was prominently higher than that in healthy individuals. *E. coli* is one of the most important pathogens in immunocompromised cases with great concern. It may demand longer hospital stays and specialized treatment modalities in order to overcome consequent bacteremia [ 23 ]. *E. coli* sepsis also causes almost 40,000 deaths per year in the United States and result in morbidity and health care finances [ 24 ].

Sharma *et al*. [ 24 ] reported a rare case of pyomyositis due to *E. coli* in immunosuppressed subject. Olson *et al*. [ 23 ] in a retrospective study evaluated E.coli bacteremia in hospitalized cases with hematopoietic malignancies. They found that E.coli is a primary pathogen in this group.

Derafshi *et al*. [ 25 ] showed that the dentures might act as reservoir for non-commensal bacteria such as Enterobacter cloacae.

A study enrolled by Back-Brito *et al*. [ 26 ] has also indicated that the oral microflora can change subsequent to immune deficiency. They reported colonization with non-commensal oral bacterial species such as *Staphylococcus aureus, Enterobacteriaceae* and *Pseudomonadaceae* in Brazilian HIV positive cases [ 26 ]. It seems that decreased CD4 cell counts might be related to this finding.

In the previous studies on oral bacterial flora of patients with leukemia, non-oral pathogenic bacteria were isolated from the oral cavity of these subjects. *Klebsiella, Enterobacteriaceae, Phylum Firmicutes, Lactobacillales, Aerococcaceae* and *Carnobacteriaceae*, Abiotrophia and Granulicatella were identified from samples. Children with leukemia established a structural difference of the oral microbiome, possibly caused by systemic infections [ 27 - 28 ]. 

These findings represent the significance of immune status in determining oral microflora and the importance of oral bacteria in inducing systemic diseases.

Leung *et al*. [ 29 ] evaluated mouth rinse samples of individuals after radiotherapy in the head and neck region, and observed that the prevalence of Enterobacteriaceae in individuals between the ages of 48 and 60 years was 32%.

In current research, there was no statistically difference between age and gender of cirrhotic cases and the control group. Similar to our results, Agwu *et al*. [ 30 ] have been stated that HIV+ cases harbor hazardous enteric bacteria in the saliva, but there was no difference between males and females. It should be noted that cross-sectional studies have a limited time which can single viewed the oral microflora. The transient oral microbiota is present in a complex dynamic environment and may occur in another occasion. Further studies with more precise method are recommended in order to gain more adequate results [ 31 ]. Experimentally, the use of mouthwashes such as chlorhexidine can reduce the load of *E.coli in vitro*; which might help the CLF cases [ 32 ].

## Conclusion

Oral cavity may act as a reservoir for enteric bacteria such as *E. coli* in cirrhotic patients. Debilitating disease may increase the risk of retention of such bacteria in the mouth. Adequate oral and general hygiene may reduce the risk of systemic infection especially in immunocompromised cases. Further studies are recommended in order to gain more accurate results.
